# A comprehensive study on bisphenol A degradation by newly isolated strains *Acinetobacter* sp. K1MN and *Pseudomonas* sp. BG12

**DOI:** 10.1007/s10532-020-09919-6

**Published:** 2020-11-17

**Authors:** Magdalena Noszczyńska, Michalina Chodór, Łukasz Jałowiecki, Zofia Piotrowska-Seget

**Affiliations:** 1 Institute of Biology, Biotechnology and Environmental Protection, Faculty of Natural Sciences, University of Silesia in Katowice, Jagiellońska 28, 40-032 Katowice, Poland; 2 Microbiology Unit, Institute for Ecology of Industrial Areas, Kossutha 6, 40-844 Katowice, Poland

**Keywords:** Bisphenol A, *Acinetobacter* sp. K1MN, *Pseudomonas* sp. BG12, Biodegradation, Toxicity

## Abstract

Bisphenol A (BPA) is an endocrine disrupting chemical. Its extensive use has led to the wide occurrence of BPA in various environmental ecosystems, at levels that may cause negative effects to the ecosystem and public health. Although there are many bacteria able to BPA utilization, only a few of them have a strong capacity for its biodegradation. Therefore, it is important to search for new bacteria strains, investigate their BPA biodegradation ability and potential effect of pH and other organic compounds on the process. These tasks have become the object of the present study. The results of our research show that for the newly isolated strains *Acinetobacter* sp. K1MN and *Pseudomonas* sp. BG12 after 15 days, with an initial BPA concentration of 100 mg L^− 1^, the highest BPA removal was achieved at pH 8, while sodium glutamate as a biostimulant best accelerated BPA degradation. Kinetic data for BPA biodegradation by both strains best fitted the Monod model. The specific degradation rate and the half saturation constant were estimated respectively as 8.75 mg L^− 1^ day^− 1^ and 111.27 mg L^− 1^ for *Acinetobacter* sp. K1MN, and 8.6 mg L^− 1^ day^− 1^ and 135.79 mg L^− 1^ for *Pseudomonas* sp. BG12. The half-maximal effective concentration (EC_50_) of BPA for *Acinetobacter* sp. K1MN was 120 mg L^− 1^ and for *Pseudomonas* sp. BG12 it was 123 mg L^− 1^. The toxicity bioassay (Microtox test) showed that elimination of BPA by both strains is accompanied by reduction of its toxic effect. The ability of tested strains to degrade BPA combined with their high resistance to this xenobiotic indicates that *Acinetobacter* sp. K1MN and *Pseudomonas* sp. BG12 are potential tools for BPA removal during wastewater treatment plant.

**Electronic supplementary material:**

The online version of this article (10.1007/s10532-020-09919-6) contains supplementary material, which is available to authorized users.

## Introduction

Bisphenol A (BPA; 2,2-bis(4-hydroxyphenyl)propane) is an endocrine-disrupting chemical (EDC) capable of interfering with the function of sex hormones, insulin, leptin, and thyroxin (García-Espiñeira et al. 2018). It can also induce immunotoxic, mutagenic, genotoxic, hepatotoxic, teratogenic, neurotoxic and carcinogenic effects, even at nanomolar level (Pfeifer et al. 2015).

Despite BPA’s negative impact on the human body, it is one of the most commonly produced and used compounds worldwide with annual production expected to reach 10.6 million metric tons in 2022. Its annual growth rate between 2016 and 2022 is approximately 4.8% (Industry Experts, 2016). Because of the wide usage of polycarbonate plastics and epoxy resins in industry and households, BPA is a prevalent contaminant in the environment and its concentration, especially in the aquatic environment, is constantly increasing (Cleveland et al. 2014; Bilal et al. 2019; Grelska and Noszczyńska 2020). It enters these ecosystems mainly through the effluents of wastewater treatment plants (WWTPs), where by lack of efficient systems of its removal, BPA may contaminate drinking water sources downstream (Zielinska et al. 2019).

Taking into account that BPA possesses an ecological risk, there is an urgent necessity to eliminate it from the environment. One of the ways to remove BPA from ecosystems is its microbial degradation. Therefore, searching for efficient BPA degraders and detailed studies on microbial utilization of BPA are vital to engineer methods that enable its effective elimination from different environments compartments. A large number of bacteria capable of BPA degradation have been isolated from different environments such as rivers, seawater, wastewaters, leachates, sludges, soil, desert soil and the rhizosphere of plants (Kang and Kondo 2002; Sasaki et al. 2005a, b; Toyama et al. 2009; Fischer et al. 2010; Zühlke et al. 2016; Kamaraj et al. 2018; Suyamud et al. 2018; Louati et al. 2019). In some of these bacteria, a few enzymes active in BPA utilization have been identified. A cytochrome P450 monooxygenase was reported to catalyze the transformation of BPA to BPA-M and the BPA ipso substitution in *Sphingomonas* sp. AO1 and *Sphingomonas* sp. TTNP3, respectively (Sasaki et al. 2005a, b; Kolvenbach et al. 2014). An ammonia monooxygenase in *Nitrosomonas europaea* and an extracellular laccase in *Pseudomonas* sp. LBC1 were also identified to be involved in BPA utilization (Kolvenbach et al. 2007; Roh et al. 2009; Telke et al. 2009). Also proteins engaged in protocatechuate transformation are probably involved in the BPA degradation pathway in *Sphingobium* sp. BiD32. Moreover, a *p*-hydroxybenzoate hydroxylase, which likely takes part in metabolism and degradation of xenobiotics, was also linked with BPA degradation by the strain BiD32 (Zhou et al. 2015).

Most of these abovementioned bacterial isolates degraded about 70% of BPA in medium containing up to 1 mg L^− 1^ of the compound (Kang and Kondo 2002; Sasaki et al. 2005a, b; Toyama et al. 2009; Fischer et al. 2010; Zühlke et al. 2016; Kamaraj et al. 2018; Suyamud et al. 2018; Louati et al. 2019). At a relatively high concentration of BPA, the efficiency of bacterial degradation decreased (Fischer et al. 2010; Kamaraj et al. 2014; Heidari et al. 2017).

Taking this into account and the constantly increasing amount of BPA in the aquatic environments, it is necessary to search for new pure bacterial strains that have potential to degrade BPA in high concentration what may lead to the development of successful biodegradation strategy of this compound which could be applied in WWTPs. However, in wastewater, apart from xenobiotics, other organic compounds are present. These compounds might served as additional growth substrates for bacteria and might affect their enzyme stability hence changing bacteria’s potential to degrade xenobiotics. Another factor which determine the enzyme stability is pH. Therefore, the main novelty of the present study was to determine how selected additional growth substrates and pH influence on BPA degradation rate by newly isolated bacteria utilizing BPA at concentration of 100 mg L^− 1^. We also estimated the kinetic parameters of the degradation process, determined the inhibitory effect of BPA on bacterial growth, and analyse BPA toxicity before and after degradation by the isolated strains.

## Materials and methods

### Reagents and media

Bisphenol A and ethyl acetate (HPLC grade) were obtained from Merck (Darmstadt, Germany). HPLC grade acetonitrile and ethanol were purchased from S. Witko - JT Baker (Lodz, Poland). Water used as a HPLC solvent was purified with a Direct-Q® Water Purification System (Merck). The standard stock solutions of BPA (7 g L^− 1^ or 5 mg 5 mL^− 1^) were prepared in 70% ethanol and stored at 4 °C up to three months. Final concentration of BPA and ethanol in medium were 100 mg L^− 1^ and 1% (v/v), respectively. Basal Salt Medium (BSM) (Badiefar et al. 2015) was used for isolation and purification of bacterial species and the degradation study. For bacteria isolation, BSM was supplemented with nystatin (4 g L^− 1^) and actidione (4 g L^− 1^) to inhibit the growth of fungi.

### Sampling and isolation of bisphenol A degrading bacteria

Samples used for isolation of BPA-degrading bacteria were collected from WWTP Klimzowiec, Katowice (1000 ml of activated sludge), a landfill in Tychy (1000 mL of leachate), Petrochemia-Blachownia SA, Kędzierzyn Koźle (10 g of soil) and Kalina pond, Świętochłowice (1000 ml of water with sediment).

**Fig. 1:**
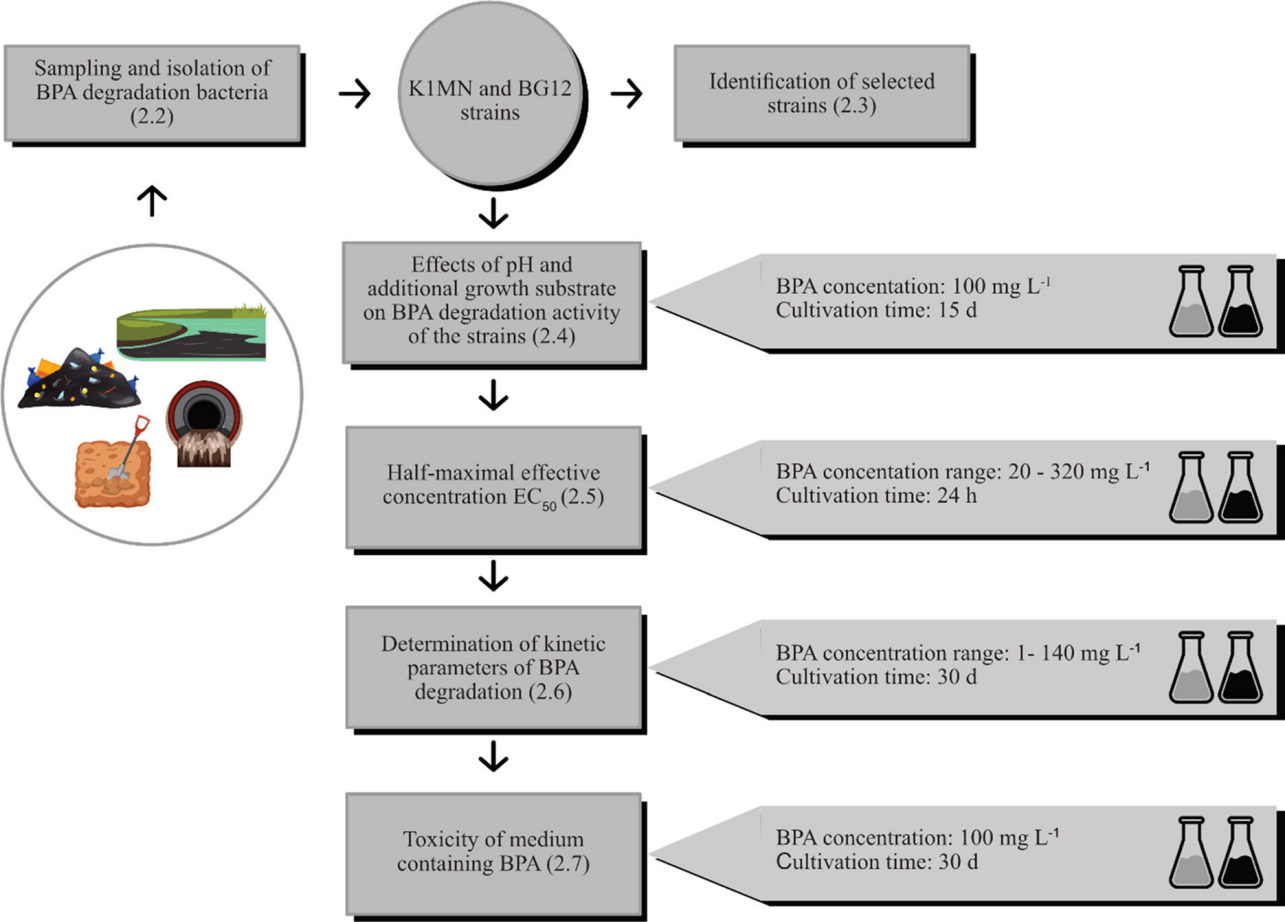
Flowchart of the experiment procedures used in this study. Erlenmeyer flask with grey content indicates the K1MN strain, and the dark one indicates the BG12 strain

The activated sludge, leachate and water with sediment were centrifuged (4700 rpm, 20 min, 4 °C). Supernatants were discarded and pellets were re-suspended in 20 mL of sterile Millipore Water. 10 mL of these suspensions and 10 g of the collected soil were added to separate flasks containing 90 mL of BSM supplemented with BPA at a final concentration of 20 mg L^− 1^ and incubated at 28 °C with rotary shaking (120 rpm). After 7 days, 10 mL of acclimated consortiums were used as inocula to start fresh batches with gradually increasing concentrations of BPA (40–100 mg L^− 1^). The morphologically distinct bacterial strains were isolated and screened for strains with the highest BPA degradation abilities. Briefly, the selected strains were cultivated in 100 mL of BSM enriched with 100 mg L^− 1^ BPA, at 28 °C and 120 rpm for six days. Then, 1 mL of each culture was collected and BPA concentration was measured using high-performance liquid chromatography (HPLC). The two most effective BPA degraders named K1MN and BG12 were selected for further experiments. The concept of these experiments is presented in Fig. 1.

### Identification of selected strains

The two selected strains K1MN isolated from Kalina pond and BG12 isolated from soil were morphologically and phenotypically characterized (API 21 system, BioMerieux, Lyon, France).

Genomic DNA was extracted from K1MN and BG12 using a DNA extraction kit (Bacterial & Yeast Genomic DNA Purification Kit, EURx) according to the manufacturer’s instructions. The 16S rRNA gene sequence was amplified with primers 8F and 1492R targeting a fragment size of 1484 bp (Pacwa-Płociniczak et al. 2014). The PCR reaction contained: 1 µL of the DNA template, 0.125 µL DreamTaq DNA polymerase (5 U/µL) (Thermo Fisher Scientific), 2.5 µL 10 × DreamTaq Buffer (Thermo Fisher Scientific), 1 µL dNTP Mix (10 mM; Thermo Fisher Scientific, Invitrogen™), 1 µL 16S Forward Primer (0.1 µg/mL), 1 µL 16S Reverse Primer (0.1 µg/mL), 25 µL reaction. PCR amplification was performed at 95 °C for 5 min, 3 cycles at 94 °C for 45 s, 57 °C for 30 s, 72 °C for 120 s; 3 cycles at 95 °C for 45 s, 56 °C for 30 s, 72 °C for 120 s; 3 cycles at 95 °C for 45 s, 56 °C for 30 s, 72 °C for 120 s; 31 cycles at 95 °C for 45 s, 53 °C for 30 s, 72 °C for 120 s; and a final elongation cycle at 72 °C for 5 min in a C1000 Touch™Thermal Cycler (BioRad). Gene sequencing was performed by an external company (Genomed, Poland). The obtained sequences (1417 bp for K1MN and 1411 bp for BG12) were compared with EZBioCloud database. The phylogenetic analysis was done based on the longest common fragment of the 16S rRNA gene sequences selected from ClustalW alignment of K1MN and BG12 strains and closest type strains of other *Acinetobacter* and *Pseudomonas* species, respectively which were obtained from GenBank (Larkin et al. 2007; Furmanczyk et al. 2018). Phylogenetic analysis and evolutionary distance calculations were determined using the maximum-likelihood method based on 1000 bootstrap resampling and a Tamura 3-parameter model assuming that a certain fraction of sites are evolutionarily invariable (+ I) conducted using Mega X software (Furmanczyk et al. 2018; Kumar et al. 2018). The determination of the similarity of 16S rRNA sequences between tested and type strains was done using the Average Nucleotide Identity (ANI) calculator (https://www.ezbiocloud.net/tools/ani) (Płociniczak et al. 2019).

### Effects of pH and additional growth substrate on BPA-degrading activity of the strains

Degradation experiments were performed in Erlenmeyer flasks containing 250 mL of BSM and BPA (100 mg L^− 1^) at pH 7.2. The flasks were supplemented with glucose, sucrose, monosodium glutamate (1 g L^− 1^) or phenol (5%) to evaluate the effect of the additional carbon sources as biostimulants on BPA degradation. Each created set was made in triplicate and inoculated either with 100 µL of the K1MN or BG12 cells being at logarithmic growth phase (log-phase cells) (OD_600_ = 0.3). As a control non-inoculated media were used. The biodegradation experiments were maintained for 15 days at 28 °C, pH 7.2 with rotary shaking, 120 rpm. Samples were collected periodically every 5 days to determine the growth of studied strains (OD_600_) and evaluate BPA concentration.

In order to study the effect of pH value on BPA degradation, 250 mL of BSM with BPA (100 mg L^− 1^) were adjusted to pH in the range 3–8. All media at given pH were made in triplicate and inoculated with log-phase cells and the experiment was carried out according to the procedure described above.

### Half-maximal effective concentration (EC_50_)

To elucidate the inhibitory effect of BPA on the growth of tested strains, log-phase cells were adjusted to the optical density 0.05 and 100 µl of the suspension was transferred to the nutrient broth supplemented with BPA in the concentration range of 20–320 mg L^− 1^. Each set was prepared in triplicate. After 24 h cultivation with rotary shaking, 120 rpm, at 28 °C, the OD_600_ of the cultures was measured. The EC_50_ value was calculated using five parameter logistic regression with SigmaPlot 14.0 software. The equation is presented below

$$y=min+\frac{max-min}{{\left[1+{\left(\frac{x}{{x}_{b}}\right)}^{-Hillslope}\right]}^{s}}$$

where

$${x}_{b}={EC}_{50}\times {10}^{\left[\left(\frac{1}{Hillslope}\right)log\left({2}^{\left(\frac{1}{s}\right)}-1\right)\right]}$$

$min$ is the bottom of the curve; $max$ is the top of the curve; $Hillslope$ characterizes the slope of the curve at its midpoint; $s$ is the asymmetry parameter; $x$ is the BPA concentration, $y$ is the optical density of the bacterial culture.

### Determination of kinetic parameters of bisphenol A degradation

For analysis of the degradation kinetics of BPA, 0.01 g of log-phase cells of K1MN and BG12 strains were separately inoculated in a series of 300 mL Erlenmeyer flasks containing 150 mL of BSM supplemented with BPA at initial concentrations of 1, 10, 30, 60, 100, 120 or 140 mg L^− 1^. This range of concentrations was selected based on the obtained results in experiment described in Sect. 2.5. Each set of flasks was prepared in triplicate. Flasks were incubated for 30 days at 28 °C with shaking at 100 rpm. Every 24 h, growth of strains was monitored by OD_600_ measurement and the concentration of residual BPA in the medium was determined. For studying biodegradation of BPA, the Monod model was used, which is presented by the following equation (Eq. 1):

1 $$SDR=\frac{VmaxS}{Ks+S}$$

where S is the substrate concentration (mg L^− 1^), *V*_*max *_is the maximum specific BPA degradation rate (mg L^− 1^ day^− 1^), *K*_*s*_ is the half saturation constant (mg L^− 1^).

Kinetic constants were estimated using SigmaPlot 12.0 software.

### Toxicity bioassay

The acute toxicity of the initial and residual BPA concentration after 30-days degradation by K1MN and BG12 strains was evaluated by the Microtox test. The initial concentration of BPA (100 mg L^− 1^) was selected on the basis of EC_50_ and *K*_*s*_ values obtained for both strains. Each setup was done in triplicate. The assay was performed in accordance with the manufacturer’s procedures using freeze-dried *Vibrio fischeri* NRRL B-11,177 and Microtox Model 500 Analyser (Modern Water Inc., UK). The inhibition of the luminescence of NRRL B-11,177 was compared to the control sample (bacteria not treated with BPA) after 5 and 15 min of exposure. The standard protocol 81.9% Basic Test was done during which all samples were diluted with 2% NaCl at 81.9% of the initial sample concentration. The toxicity units (TU = 1/EC_50_ ^− 1^ × 100) were calculated using the MicrotoxOmni (Microbics Corp, 1992) program (Le et al. 2017).

### Analytical methods

For the determination of BPA concentration, 1 mL of cultures was collected and centrifuged (14,000 rpm, 10 min). 1 mL of ethyl acetate was added to the resultant supernatants and the mixtures were vortexed at 3000 rpm for 60 s followed by centrifugation (1000 rpm, 60 s). Organic layers were transferred to Pyrex glass and dried under a stream of N_2_. 1 mL of 70% ethanol was added to the pellets and samples were vortexed at 3000 rpm for 60 s. Solutions were filtered through 0.2 µm RC membrane filter (Hahnemuehle, Germany) and used for detection and measurement of BPA content using a Shimadzu HPLC (Kyoto, Japan) with quaternary pumps (model LC-20AD), connected to a PDA detector (Shimadzu, model SPD-M20A) interfaced with the LabSolutions software. Separation was achieved using the Phenomenex Synergi 4 µm Hydro-RP (150 × 4.6 mm) column, protected by an AQ C18 guard column (Phenomenex, Torrance CA, USA). The samples were eluted with a linear gradient of acetonitrile–water (90−10%) with a flow rate of 1 mL min^− 1^ for 35 min. The column temperature was maintained at 30 °C. The injection volume was 15 µL. All experiments were carried out in triplicate and results are expressed as an average value.

The calibration curve was obtained from a linear regression program by concentrations versus detector responses using concentration levels for eight standards. These working solutions were prepared from stock solution of 1 g L^− 1^ at concentrations of 0.5, 10, 50, 100, 200, 400, 600 and 1000 mg L^− 1^. The correlation coefficient of peak height to concentration was > 0.998.

The percentage BPA removal efficiency (RE) was calculated using the equation:

$$\% RE=\frac{(Co-Cf)}{Co} \times 100$$

where Co and Cf are the initial and final concentrations of BPA (mg L^− 1^) in BSM, respectively.

### Statistical analysis

In order to check the normality of the data for all of the results obtained in Sects. 2.4 and 2.7, the Shapiro-Wilk tests was used. A one-way or two-way ANOVA analysis (p < 0.05) followed by a Fisher’s least significant difference (LSD) test was performed to conduct the statistical significance. Statistical analysis was done using STATISTICA 13.1 PL software (StatSoft, Tulsa, USA).

## Results and discussion

### Isolation and identification of selected strains

Forty bacterial strains able to grow in the presence of 100 mg L^−1^ BPA as a sole carbon source were isolated after 5 weeks of incubation. The BPA degrading activities of these strains were determined as a loss of BPA (100 mg L^−1^) amount in BSM after 6 days by HPLC analysis. All isolates were able to grow in the medium and degraded BPA with different efficiency (data not shown). For two strains named K1MN and BG12 the RE was higher than to the other strains and was 20 ± 3% and BG12 36 ± 2%, respectively. These strains were isolated from Kalina pond (K1MN) and soil from Petrochemia-Blachownia SA area (BG12).

The strains were characterized by morphological and biochemical assays (Table 1 in supplementary data). The bacteria were rod-shaped and Gram-negative. Strain K1MN similar to *Acinetobacter johnsonii* was positive for citrate utilization and negative for gelatin hydrolysis as well as assimilation of glucose and arabinose (Kozińska et al. 2014; Juni 2015). Strain BG12 assimilated glucose and was negative for nitrate reduction, analogously to *Pseudomonas protegens* strain CHA0 (Ramette et al. 2011).

**Fig. 2:**
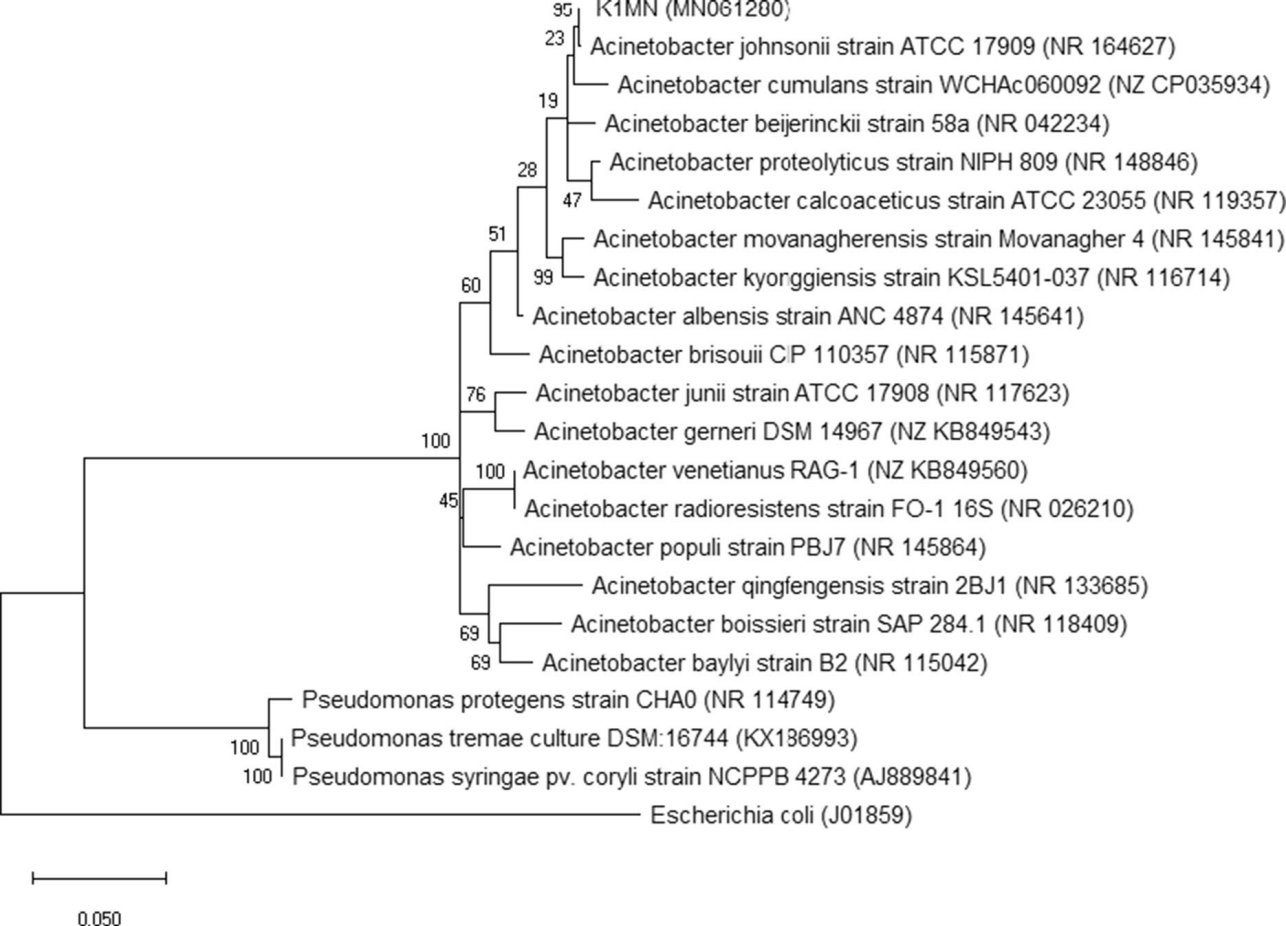
Phylogeny of type strains closely related to K1MN strain based on 16S rRNA gene sequence. All positions containing gaps or missing data were eliminated, which resulted in a 1283 bp sequence in the final dataset. Bootstrap values are represented at the branching points. The bar represents 0.05 substitutions per site. Accession numbers of sequences used in this analysis are in parentheses

The phylogenetic analysis based on the ClustalW alignment of the 16S rRNA gene sequences of strain K1MN (1283 bp) and the 17 closest type strains of other *Acinetobacter* species placed it in the *A. johnsonii* subgroup (Fig. 2).

**Fig. 3:**
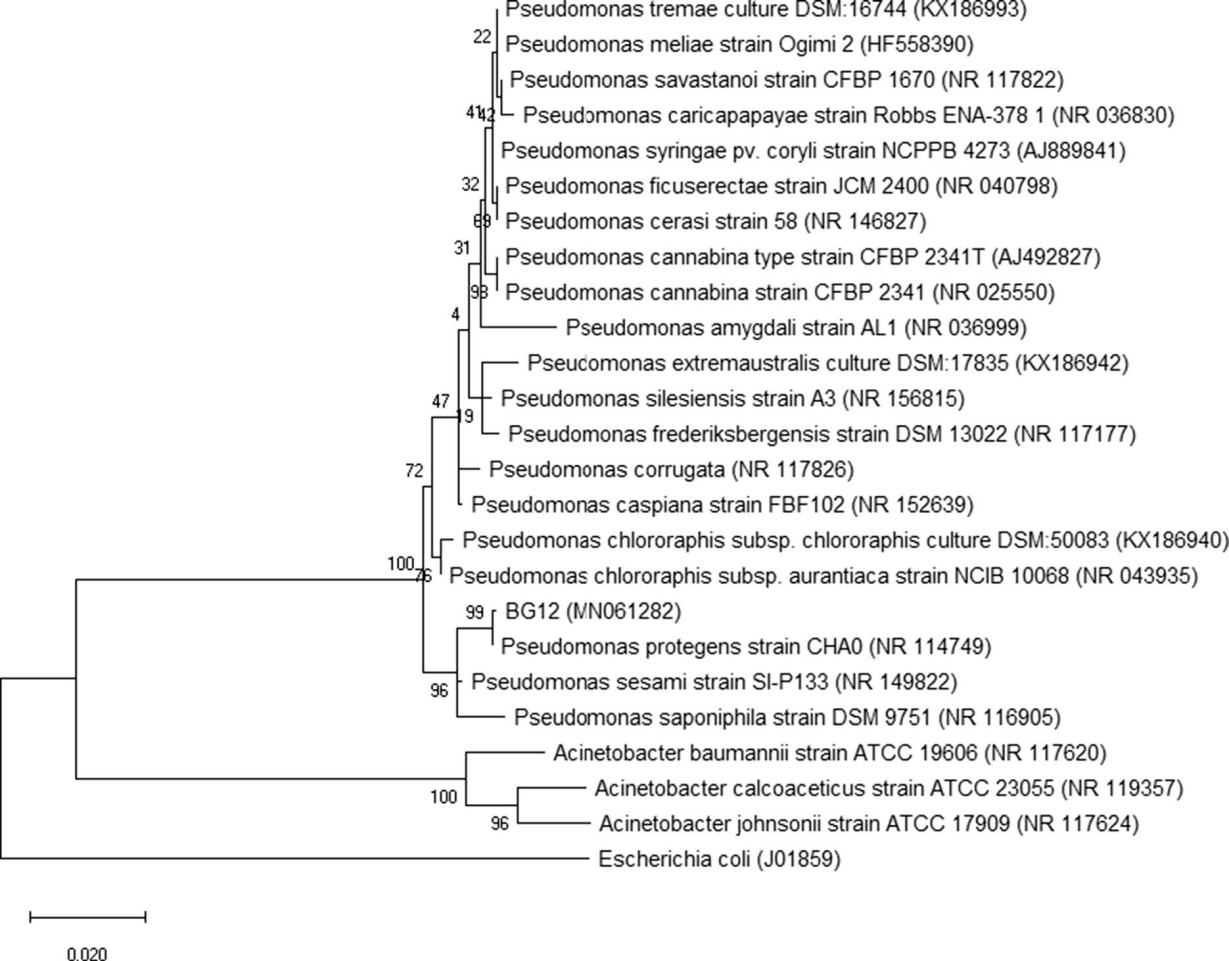
Phylogeny of type strains closely related to BG12 strain based on 16S rRNA gene sequence. All positions containing gaps or missing data were eliminated, which resulted in a 1333 bp sequence in the final dataset. Bootstrap values are represented at the branching points. The bar represents 0.020 substitutions per site. Accession numbers of sequences used in this analysis are in parentheses

The analysis of the BG12 sequence (1333 bp) and its 20 closest type *Pseudomonas* strains revealed that it is clustered in the *P. protegens* subgroup (Fig. 3). To verify the phylogenetic relationship of isolated strains, the ANI was calculated among them and related species. These results confirmed that the closest related species of strain K1MN is *A. johnsonii* ATCC 17,909 with similarity of 99.10%. The next closest related species are: *A. bouvetii* DSM 14,964 with 98.20%, *A. lwoffi* DSM 2403 with 96.80%, *A. kyonggiensis* KSL5401-037 with 96.65%, and *A. albensis* ANC 4874 with 95.70%. In the case of the BG12 strain, the sequence of the 16S rRNA gene showed 99.90% similarity with *P. protegens* CHA0, 99.0% similarity with *P*. *sesame* SI-P133 and 98.40% similarity with *P*. *saponiphila* DSM 9751.

Based on morphologic, phenotypic, ANI and partial 16S rRNA analyses, isolates were identified as *Acinetobacter* sp. strain K1MN and *Pseudomonas* sp. strain BG12.

### Effects of pH and additional growth substrate on BPA-degrading activity of the strains

As it has been mentioned before, *Acinetobacter* sp. K1MN and *Pseudomonas* sp. BG12 were able to degrade 20 ± 3% and 36±%2 BPA at an initial concentration of 100 mg L^− 1^, respectively within 15 days. Since pH values strongly influence the activity of most enzymes, their impact on BPA degradation capacity of the strains was studied. Moreover, in the wastewaters besides from xenobiotics other various organic compounds are present. On the one hand, some of them might blocking the active sites of degradation enzymes thus inhibiting degradation processes (Górny et al. 2019). On the other hand, some organic compounds contribute to the growth of bacterial cells and the faster degradation of xenobiotics by being as an additional carbon source for bacteria and/or by producing specific monooxygenase enzymes by the bacteria in the case of the structural analogue of the degraded pollutant (Domaradzka et al. 2015; Górny et al. 2019). Therefore, it was interesting to verify whether pH and additional growth substrate such as phenol, glucose, saccharose and sodium glutamate can affect the degradation capacity of the tested strains.

**Fig. 4:**
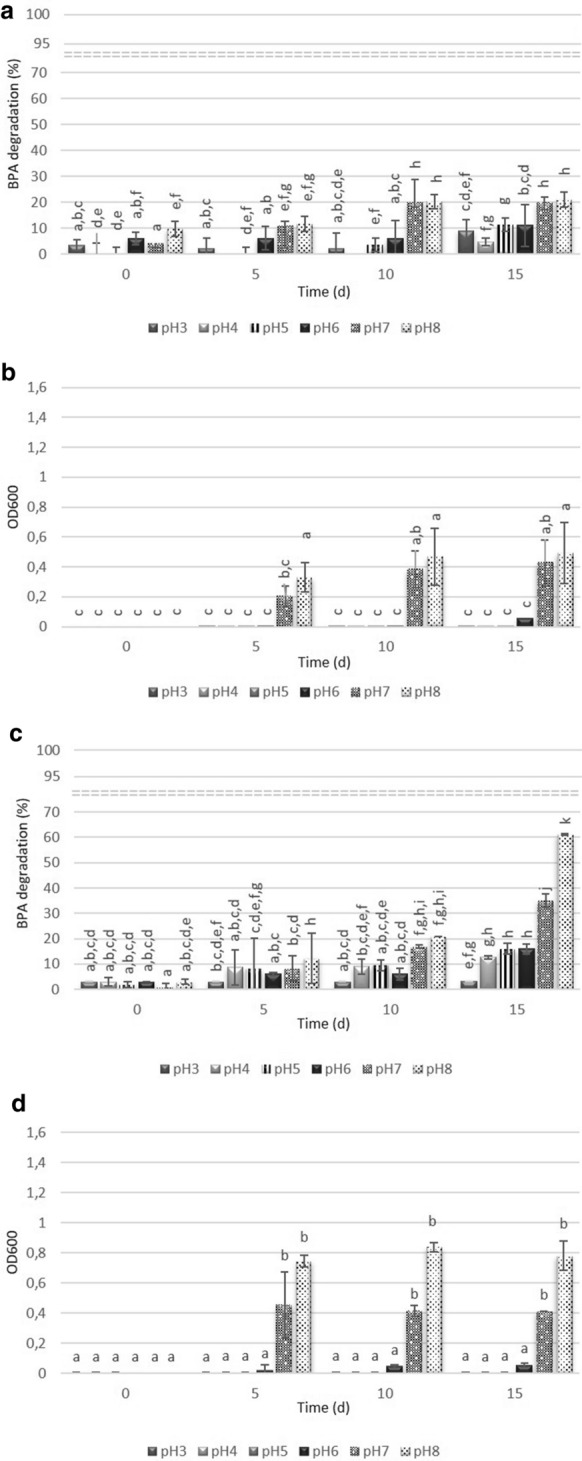
Degradation efficiency (*Acinetobacter* sp. K1MN – a, *Pseudomonas* sp. BG12 – c) and strains’ growth monitored as optical density at 600 (*Acinetobacter* sp. K1MN – b, *Pseudomonas* sp. BG12 – d) in BSM medium with BPA (100 mg L^− 1^). The data points represent the average of three independent experiments ± standard deviation. The same letter(s) above the bars indicate no statistical significance (MANOVA followed by Fisher’s LSD test) related to the effects of BPA degradation and optical density of cultures at *p* < 0.05

Figure 4 shows the degradation trends of BPA in the pH range from 3 to 8. For both strains, complete BPA degradation was not achieved at any of the pH levels. At pH from 3 to 6 the RE of BPA was 4–11% and 4–16% for *Acinetobacter* sp. K1MN and *Pseudomonas* sp. BG12, respectively. It was associated with a low survival rate of the strains in an acidic environment, resulting in reduced activity of degrading enzymes or even their lack (Li et al. 2012). The highest degradation values of 60% for pH 8 and 35% for pH 7 were obtained for *Pseudomonas* sp. BG12. In contrast, under these pH values a slight BPA reduction (21%) was observed for *Acinetobacter* sp. K1MN. Under weak alkaline conditions, the growth of bacteria was not inhibited and BPA was more soluble due to its hydrolysis. Moreover, at these pH values, the surface of the bacterial cells may have been negatively charged, leading to changes in electrostatic interaction between BPA and the biomass surface (Wolski et al. 2006). Such conditions result in higher BPA degradation efficiency (An et al. 2011)

These findings differ from those of Li et al. (2012), who demonstrated that *Bacillus* sp. GZB degraded BPA in 96 h in Luria-Bertani medium at pH 6.5, 7.0, 8.0, 8.5 and 9.0 with efficiencies of 87.5%, 100%, 100%, 97.7% and 81.6%, respectively. Such high RE resulted from the low initial BPA concentration (10 mg L^− 1^) and use of a rich medium. At the same initial BPA concentration but in mineral medium at pH 7, strains *Pseudomonas* sp. K-8, K-6 and KU-3 degraded BPA in 12 days with RE 81%, 78% and 74%, respectively (Kamaraj et al. 2014). By contrast, Heidari et al. (2017) observed that *Ralstonia eutropha* was not able to complete BPA removal even at low concentrations ranging from 1 to 20 mg L^− 1^ in mineral medium at pH 7. After 12 days, the BPA RE was 15–56%. Overall, these results showed, that the most suitable BPA concentration for its effective degradation is up to 10 mg L^− 1^ in rich medium at pH of 7 or 8. This is in agreement with previous studies showing that medium composition may play an important role in complete BPA removal (Badiefar et al. 2015).

**Fig. 5:**
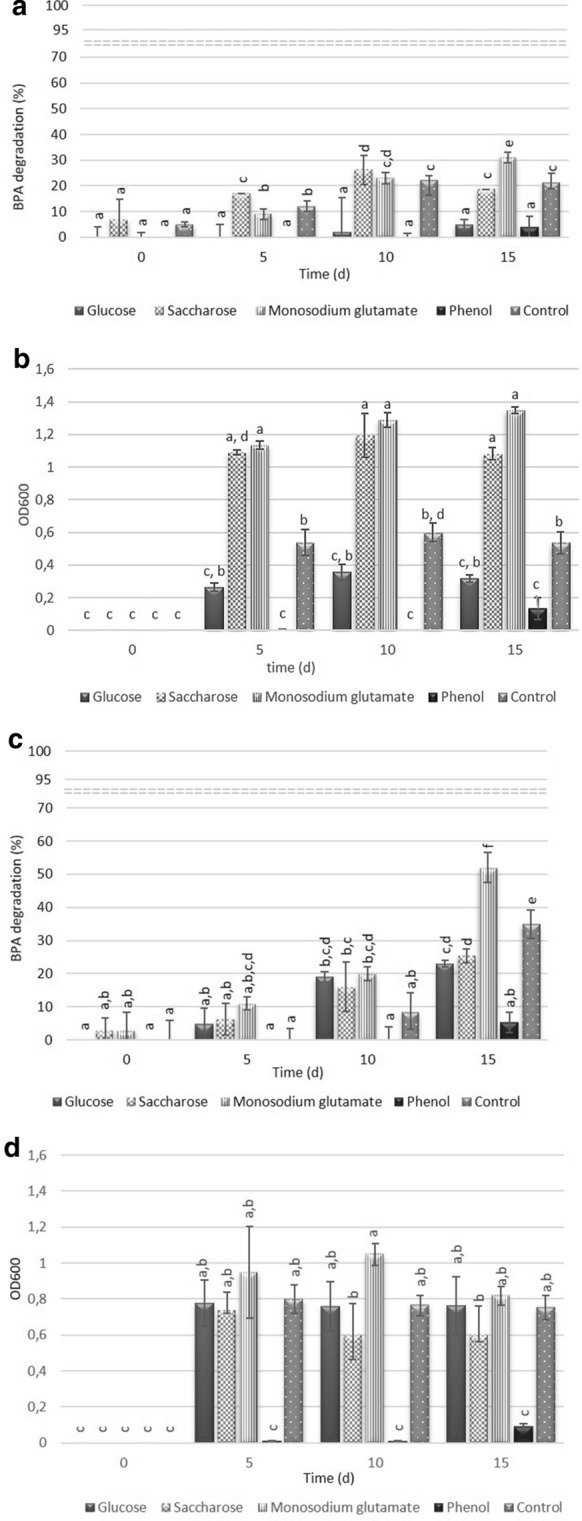
Degradation efficiency (*Acinetobacter* sp. K1MN - a, *Pseudomonas* sp. BG12 - c) and strains growth monitored as optical density at 600 (*Acinetobacter* sp. K1MN - b, *Pseudomonas* sp. BG12 - d) in BSM medium with BPA (100 mg L^− 1^) and in the presence of different, additional substrates. Controls contained no additional source of carbon. The data points represent the average of three independent experiments ± standard deviation. The same letter(s) above the bars indicate no statistical significance (MANOVA followed by Fisher’s LSD test) related to the effects of BPA degradation and optical density of cultures at *p* < 0.05

In Fig. 5 the degradation efficiency of BPA by the two tested strains in conditions with additional carbon source was shown. Due to the chemical structure similarity of phenol and BPA, it was likely that phenol would induce synthesis of the enzymes engaged in aromatic ring fission (Heidari et al. 2017). Glucose, saccharose and sodium glutamate proved to accelerate contaminant removals (Kamaraj et al. 2014; Zhao et al. 2014; Marchlewicz et al. 2017; Górny et al. 2019). However, in our study glucose and phenol caused inhibition of both BPA degradation and bacterial growth of *Acinetobacter* K1MN (Fig. 5a, b). These findings are not consistent with results reported by others who have shown that phenol stimulated growth of *Cupriavidus basilensis* JF1 but not BPA degradation, while BPA removal by *Bacillus* sp. GZB was enhanced by adding glucose (Fisher et al. 2010; Xiong et al. 2017). On the other hand, BPA RE decreased when *R. eutropha* was grown in the presence of phenol, while glucose did not affect the degradation rate of *Pseudomonas* sp. strains KU1 and KU2 as well as *Bacillus* sp. KU2 (Kamaraj et al. 2014; Babatabar et al. 2019). Similarly, saccharose did not have an influence on BPA degradation by *Acinetobacter* K1MN.

**Fig. 6:**
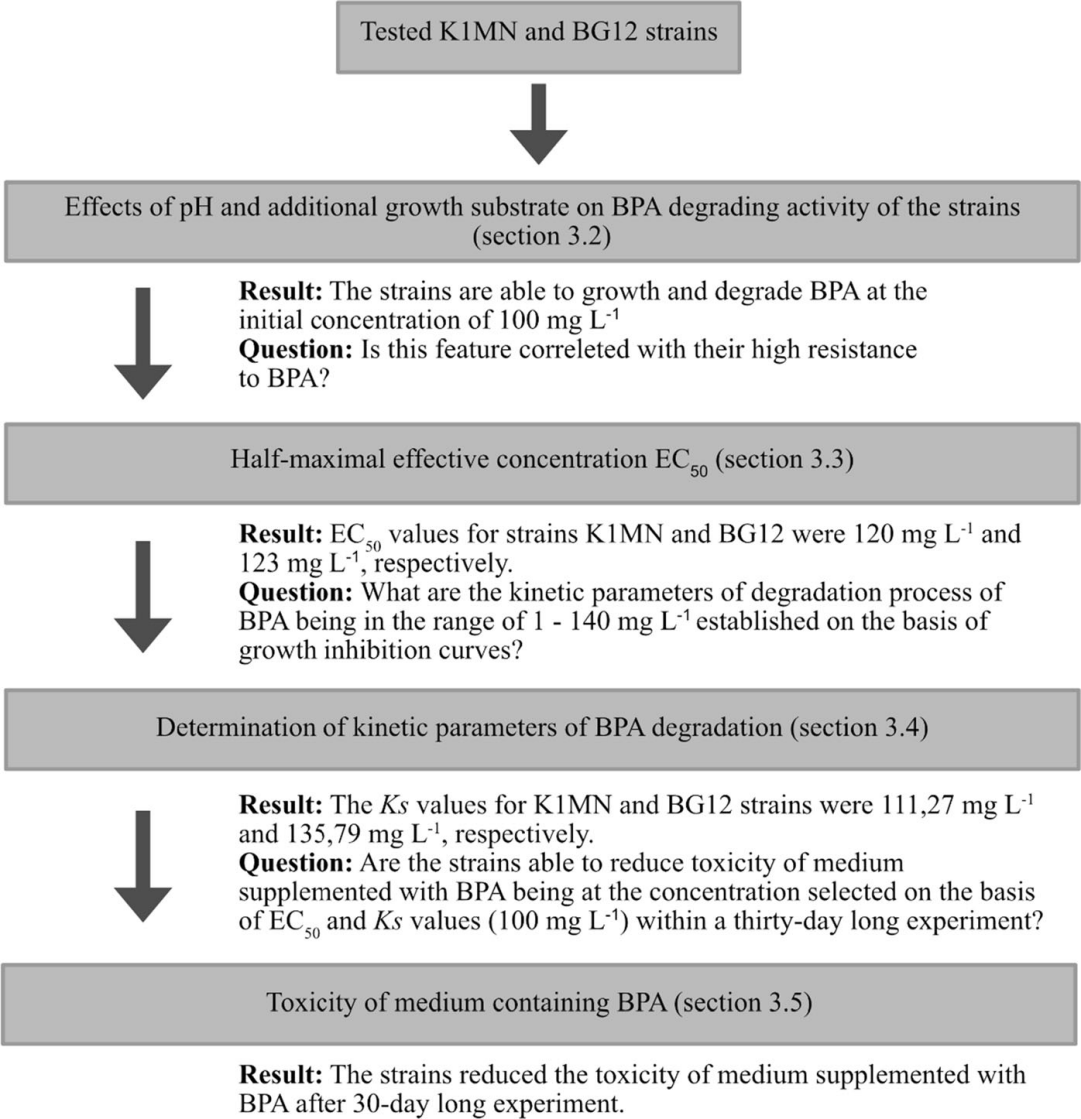
Flowchart showing how the results of individual experiments determined the conditions of subsequent analysis

The highest BPA RE was observed in the presence of sodium glutamate and reached the value of 31 ± 2.12% and 52 ± 4.58% for *Acinetobacter* sp. K1MN and *Pseudomonas* sp. BG12, respectively (Fig. 4). The addition of the substrate had a positive effect on BPA degradation efficiency in comparison to monosubstrate culture, where only 21 ± 4% (*Acinetobacter* sp. K1MN) and 35 ± 4.24% (*Pseudomonas* sp. BG12) of BPA was degraded after 15 days. We assume that the presence of sodium glutamate in the culture medium increased the tolerance of the strains to high BPA concentrations more than other tested substrates by providing a good source of readily metabolizable carbon and nitrogen to support cell growth. The findings are directly in line with previous findings. Kamaraj et al. (2014) showed that in the presence of sodium glutamate degradation of phenol by *Pseudomonas* sp. JN-6 increased by 10% while BPA RE of *Pseudomonas* sp. K-8 increased to 90%. The addition of glucose and sucrose caused a statistically significant decrease of BPA RE in the case of *Pseudomonas* sp. K1MN, while phenol completely inhibited BPA degradation by this strain (Fig. 5c, d). The negative effects of phenol on the BPA biodegradation process as well as biomass growth of both strains probably result from its toxicity and not adapting the tested strains to grow in its presence. From the presented results, it is clear that both tested strains were able to survive and utilize BPA at the concentration of 100 mg L^− 1^. Therefore, further studies (Fig. 6) were undertaken to better characterize the ability of both strains to degrade BPA.

### Half maximal effective concentration (EC_50_)

**Fig. 7:**
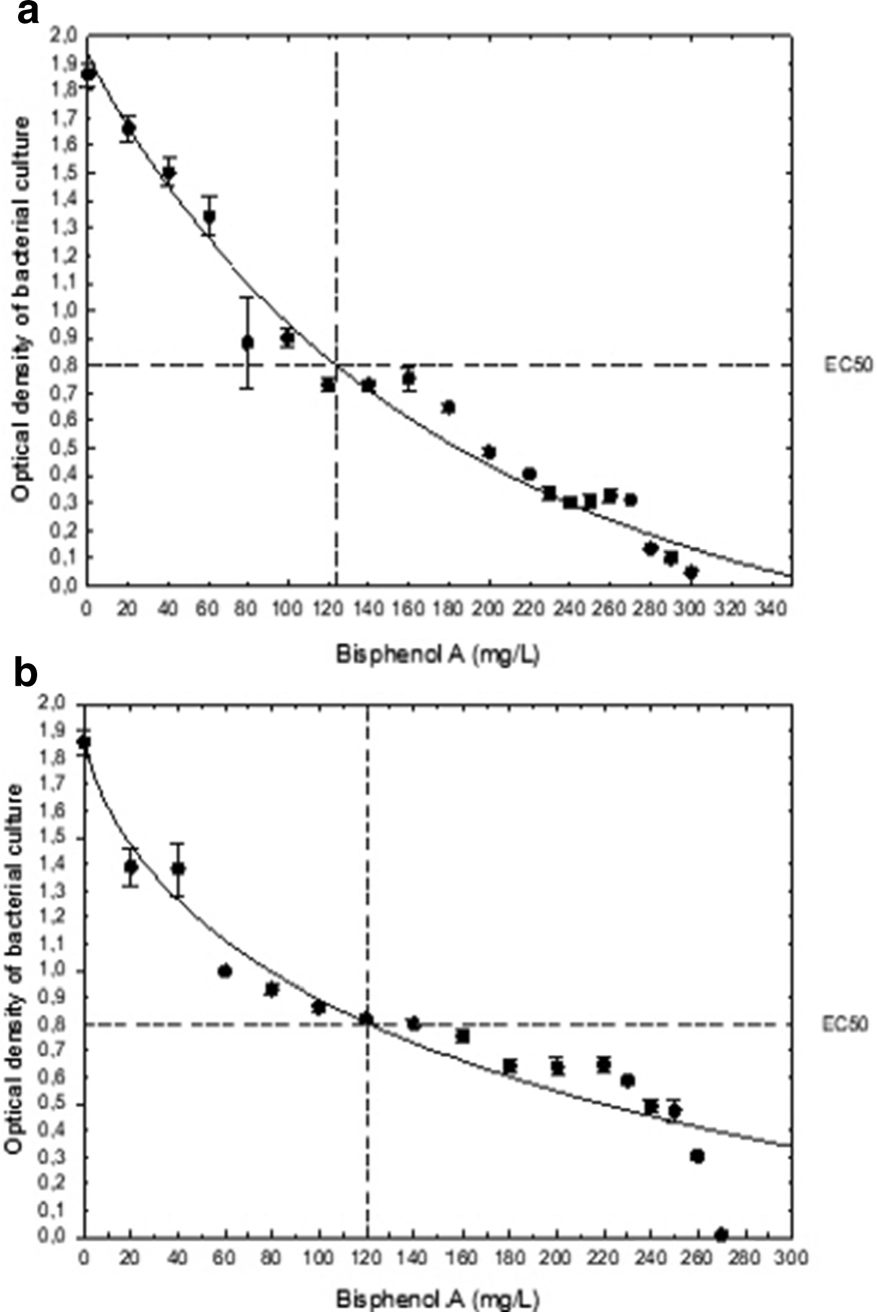
Inhibition of (a) *Acinetobacter* sp. K1MN and (b) *Pseudomonas* sp. BG12 growth in the presence of different BPA concentrations. Results shown are means ± standard deviation and the fitted 5-parameter logistic regressions in dependence of BPA concentrations in medium

In order to analyse if ability of K1MN and BG12 strains to degrade BPA at the concentration of 100 mg L^− 1^ is correlated with the higher resistance of these strains to the compound, EC_50_ values were calculated based on growth inhibition curves by various concentrations of BPA. Effective concentration in this case means the BPA concentration causing 50% growth inhibition of particular strain. Both used strains tolerate relatively high BPA concentrations. EC_50_ values of *Acinetobacter* sp. K1MN and *Pseudomonas* sp. BG12 were 120 mg L^− 1^ and 123 mg L^− 1^ BPA, respectively (Fig. 7). The concentration of the compound that completely inhibited growth of *Acinetobacter* sp. was 270 mg L^− 1^. *Pseudomonas* sp. BG12 tolerates higher concentrations of BPA and its growth was totally inhibited by 300 mg L^− 1^ BPA in nutrient broth medium. The results show that tested strains tolerate high BPA concentrations. It is probably caused by adaptation of the previous strain to the presence of BPA (100 mg L^− 1^) in medium and its capacity for BPA degradation. Probably as with phenol, an increase in BPA leads changes in the cell membrane and protect the cell from its toxic effects (Murínová and Dercová 2014). To compare, the EC_50_ value for *Cupriavidus basilensis* JF1 was established as 0.12 mM, equivalent to 27.39 mg L^− 1^ (Fischer et al. 2010). Reports regarding higher water organisms indicate stronger toxicity of BPA. For example, EC_50_ after 24 h of *Daphnia magna* exposure to BPA was estimated as 8.9 mg L^− 1^ (Tišler et al. 2016) and for the microalga *Cyclotella caspia*, 96 h EC_50_ was determined as approximately 8 mg L^− 1^ BPA (Li et al. 2008).

### Determination of kinetic parameters of BPA degradation

**Fig. 8:**
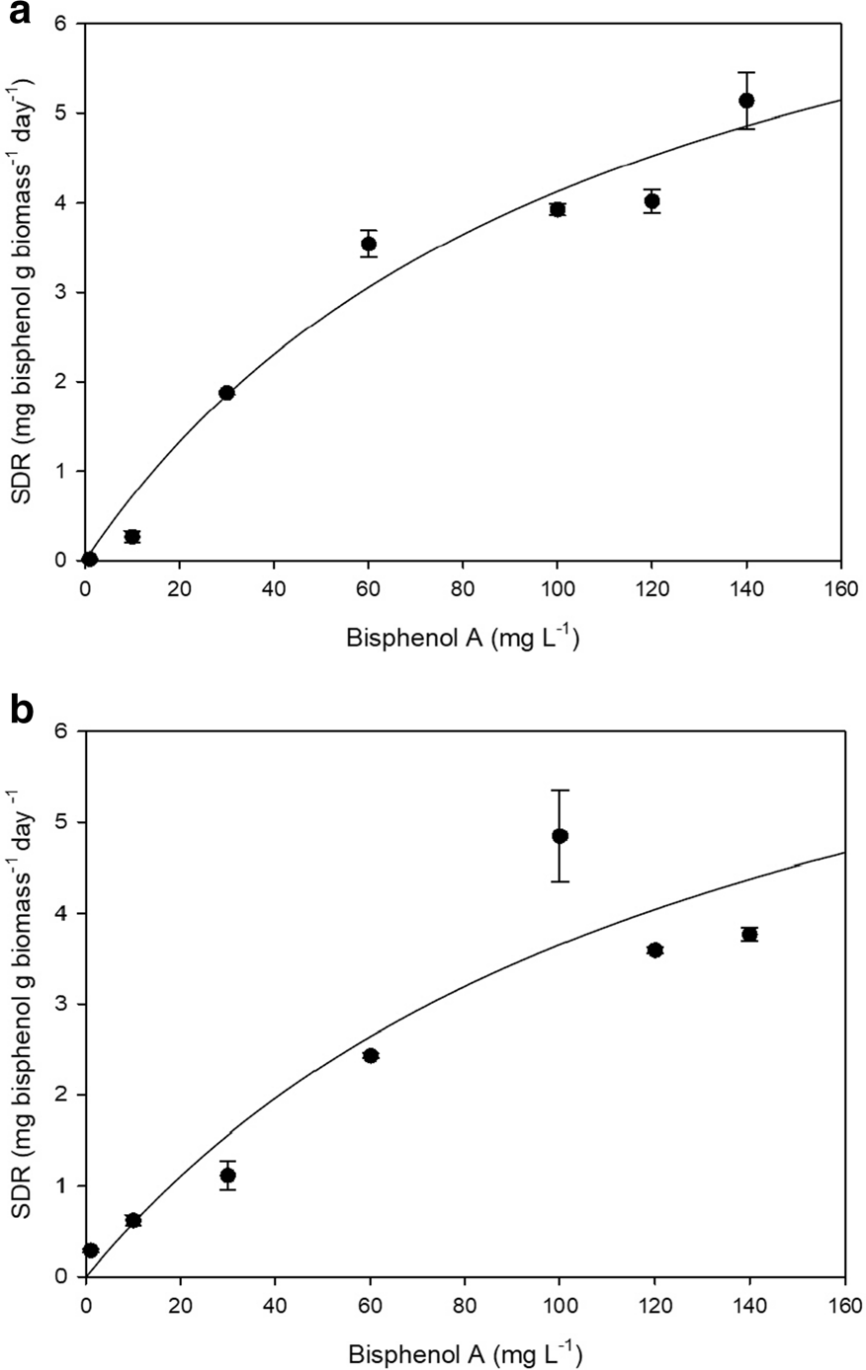
Specific degradation rate (SDR) of *Acinetobacter* sp. K1MN (**a**) and *Pseudomonas* sp. BG12 (**b**) for various BPA concentrations. The data points represent the average of three independent experiments ± standard deviation

For calculating the specific degradation rate (SDR) for *Acinetobacter* sp. K1MN and *Pseudomonas* sp. BG12, BPA concentration in a series of separate flask containing different BPA concentrations from 1 to 140 mg L^− 1^ with the initial biomass concentration of 0.01 g L^− 1^ was monitored throughout the thirty-day experiment (Babatabar et al. 2019). SDR was determined by dividing the degradation rate by the initial biomass for each initial BPA concentration. Figure 8 shows the obtained SDRs values. Different kinetic models were used to fit the experimental data (Robinson and Tiedje 1983; Okpokwasili and Nweke 2006). Among them, the Monod model gave the best fit with R^2^ = 0.97 for *Acinetobacter* sp. K1MN and R^2^ = 0.84 for *Pseudomonas* sp. BG12. Therefore, this model was used, however, the fit is not clear hence the *V*_*max*_ and *K*_*s*_ values (Table 1) might be imprecise.

**Table 1:** Comparison of kinetic constant values in BPA removal by tested strain

Kinetic constant	*Acinetobacter* sp. K1MN	*Pseudomonas* sp. BG12
V_max_ (mg L^−1^ day^−1^)	8.75	8.6
K_s_ (mg L^−1^)	111.27	135.79

The Monod equation for BPA biodegradation by *Acinetobacter* sp. K1MN can be represented by Eq. 2, and by *Pseudomonas* sp. BG12 by Eq. 3.

2 $$\frac{ds}{dt}=-SDR.X= \frac{VmaxS}{Ks+S}=\frac{8.75SX}{111.27+S}$$

3 $$\frac{ds}{dt}=-SDR.X= \frac{VmaxS}{Ks+S}=\frac{8.6SX}{135.79+S}$$

*X* represents the biomass concentration (g L^− 1^).

The *Vmax* values obtained for both strains are comparable to that of *Ralstonia eutropha* adapted for the growth of 20 mg BPA where *Vmax* = 7.4 mg L^− 1^, but significantly different from the results of *Vmax* = 0.46 mg L^− 1^ obtained for *Sphingomonas paucimobilis* FJ-4 (Fujiwara et al. 2016; Heidari et al. 2017). Thus, it is concluded that both *Acinetobacter* sp. K1MN and *Pseudomonas* sp. BG12 show great ability in BPA degradation.

### Toxicity of medium containing BPA

Even though BPA has been detected at concentrations ranging from nanograms per liter (ng L^− 1^) to micrograms per liter (mg L^− 1^) in drinking water, the long-term continuous exposure of a living organism to this EDC cannot be ignored considering its harmful impact (Sarma and Lee 2018). Therefore, it was interesting to estimate the toxicity of medium containing BPA (100 mg L^− 1^) and this medium inoculated with strains K1MN and BG12 after a 30-day cultivation period. For this purpose a Microtox bioassay, a sensitive system standardized for water and effluent samples, was chosen for toxicity evaluation (Lei and Aoyama 2010). Toxicity of each sample was determined as toxicity units (TU). An increase in TU value corresponds to an increase in toxicity (Biedroń et al. 2016). The medium with BPA after 5 and 15 min contact with *V. fischeri* showed high acute toxicity, being TU_50_ = 125.35 ± 1.7 and TU_50_ = 124.75 ± 1.2, respectively (Fig. 2 in supplementary data). After 30 days of degradation by *Acinetobacter* sp. K1MN, toxicity of medium with BPA was reduced to TU_50_ = 52.96 ± 10.99 and TU_50_ = 60.31 ± 14.13, respectively after 5 and 15 min of exposure. In contrast, metabolic activity of *Pseudomonas* sp. BG12 led to reduction of this medium toxicity to TU_50_ = 8.83 ± 2.05 after 5 min and TU_50_ = 10.34 ± 4.13 after 15 min of exposure. This result has been supported by HPLC analysis which revealed that *Pseudomonas* sp. BG12 removed 15% more of the initial BPA concentration than *Acinetobacter* sp. K1MN (data not shown). The differences in media toxicity between the two strains are probably due to the higher RE of BPA degradation by BG12 than by K1MN. This result is relevant as it demonstrates that the strains, especially *Pseudomonas* sp. BG12, are able to degrade BPA, which is correlated with a significant decrease in toxicity of the medium. It corresponds well with the research of Ike et al. (2002), who revealed that biodegradation can remarkably reduce the toxic effects of BPA. However, the main limitation of the cited study was the use of commercially available end products of one of the BPA degradation pathways, while it can be biological decomposed during various mechanisms (Noszczyńska and Piotrowska-Seget 2018). Conversely, others have shown that even when organisms degrade BPA with high RE, intermediates formed during degradation are often more toxic than the parent compound (Mtibaà et al. 2018). Similarly, chemical technologies such as photocatalytic oxidation did not reduce BPA toxicity but even increased it in comparison with untreated BPA solution (Plahuta et al. 2014).

## Conclusion

BPA is one of the most abundant pollutants in the aquatic environment and can affect surface and groundwater systems. Due to its negative impact on living organisms, it is very important to select microorganisms with the ability to decompose BPA. *Acinetobacter* sp. K1MN and *Pseudomonas* sp. BG12 seem to fulfil this expectation. Both strains showed the capacity for BPA removal. Biodegradation of this EDC was enhanced in the alkaline conditions and in the presence of monosodium glutamate. Simultaneously, other additional substrates had no positive effect on degradation ability of the strains. Promising application of the examined strains in the treatment of BPA contaminated water is related to their tolerance of high BPA concentrations and significant reduction of its toxicity. Moreover, the data from our investigation provide insight into the influence of environmental factors on BPA elimination from ecosystems.

## Electronic supplementary material

Below is the link to the electronic supplementary material.

(DOCX 22 kb)
