# Supplementary material for: A comprehensive study on bisphenol A degradation by newly isolated strains Acinetobacter sp. K1MN and Pseudomonas sp. BG12
Source: Biodegradation. 2020 Nov 17;32(1):1–15. doi: 10.1007/s10532-020-09919-6 (PMC7940318; doi:10.1007/s10532-020-09919-6)
Supplement: Supplementary file 1 — (DOCX 22 kb) [file 10532_2020_9919_MOESM1_ESM.docx]

**A comprehensive study on bisphenol A degradation by newly isolated strains *Acinetobacter* sp. K1MN and *Pseudomonas* sp. BG12**

Magdalena Noszczyńska^a*^, Michalina Chodór^a^, Łukasz Jałowiecki^b^, Zofia Piotrowska-Seget^a^,

**Table 1**. Most important phenotypical properties of K1MN and BG12

| Characteristics | K1MN | BG12 |
| --- | --- | --- |
| Nitrate reduction | + | + |
| Indole production | - | - |
| Glucose fermentation | + | + |
| Arginine dihydrolase | - | - |
| Urease | - | - |
| Hydrolysis of esculin | - | - |
| Hydrolysis of gelatin | - | + |
| β-galactosidase | - | - |
| Oxidase | + | + |
| Assimilation of: | | |
| Adipate | + | + |
| Arabinose | - | - |
| Citrate | + | + |
| Decanoic acid | - | + |
| Gluconate | + | + |
| Glucose | - | + |
| Malate | + | + |
| Maltose | - | - |
| Mannitol | + | + |
| Mannose | - | - |
| N-acetyl-L-glucosamine | + | + |
| Phenylacetate | - | - |

|  |
| --- |

**Figure 2.** Toxicity of BPA before and after degradation by *Acinetobacter* sp. K1MN and *Pseudomonas* sp. BG12 evaluated by Microtox test. The data points represent the average of three independent experiments ± standard deviation. The same letter(s) above the bars indicate no statistical significance (MANOVA followed by Fisher’s LSD test related to the decrease in BPA toxicity at *p*<0.05
